# SIRT4 is an independent prognostic factor in bladder cancer and inhibits bladder cancer growth by suppressing autophagy

**DOI:** 10.1186/s13008-023-00091-w

**Published:** 2023-06-10

**Authors:** Jie Yin, Guohao Cai, Huaiwen Wang, Weijia Chen, Shan Liu, Guoyu Huang

**Affiliations:** 1 Department of Anorectal Surgery, Suzhou Ninth People’s Hospital, Suzhou, 215200 China; 2 Department of Anorectal Surgery, Hainan General Hospital, Haikou, 570100 China

**Keywords:** Bladder urothelial carcinoma, Sirtuin, SIRT4, Prognosis, Apoptosis, Autophagy

## Abstract

**Background:**

Nucleosome-localized sirtuin 4 (SIRT4) was found to function as an oncogene and tumor suppressor gene in different tumors. However, the clinical significance of SIRT4 in bladder urothelial carcinoma (BLCA) has not been assessed, nor has the function of SIRT4 in BLCA been analyzed.

**Methods:**

In this study, we assessed the levels of SIRT4 protein in BLCA tissues and its association with clinicopathological parameters and overall survival time of BLCA patients by immunohistochemical staining of tissue microarrays containing 59 BLCA patients. Then, we constructed BLCA cell lines (T24) with overexpression or interference of SIRT4 by lentiviral infection. The effects of SIRT4 on the proliferation, migration and invasive ability of T24 cells were investigated using cell counting kit-8 (CCK-8) assays, wound healing assays, and migration and invasion assays. Moreover, we also investigated the effect of SIRT4 on the cell cycle and apoptosis of T24 cells. Mechanistically, we explored the relationship between SIRT4 and autophagy and its role in the inhibition of BLCA.

**Results:**

We found by immunohistochemistry that SIRT4 protein levels were reduced in BLCA and that lower SIRT4 levels were associated with larger tumor volumes, later T-staging and later AJCC staging in BLCA patients and were an independent prognostic factor in BLCA patients. Overexpression of SIRT4 significantly inhibited the proliferative viability, scratch healing capacity, migratory capacity, and invasive capacity of T24 cells, while interference with SIRT4 had the opposite effect. Moreover, overexpression of SIRT4 significantly inhibited the cell cycle and increased the apoptosis rate of T24 cells. Mechanistically, SIRT4 inhibits BLCA growth by suppressing autophagic flow.

**Conclusions:**

Our study suggests that SIRT4 is an independent prognostic factor for BLCA and that SIRT4 plays a tumor suppressor role in BLCA. This suggests a potential target for SIRT4 in the diagnosis and treatment of BLCA.

## Introduction

Bladder cancer (BCa) is the most common malignancy of the urinary tract. In 2018, 549,393 patients were diagnosed with BCa worldwide, while 199,922 died from the disease [1]. Of these, uroepithelial (transitional cell) carcinoma is the most common type of bladder cancer, accounting for approximately 90% of cases [2]. Surgical resection is the primary treatment for bladder urothelial carcinoma (BLCA), but the 5 year survival rate for patients with muscle-invasive BLCA is approximately 66% after surgery [3]. Therefore, a better understanding of the underlying molecular mechanism of BLCA is highly desirable for the development of effective therapeutic targets.

The SIRT family (SIRT1-7) represents a group of NAD^+^-dependent deacetylases and ADP-ribosyltransferases that play important roles in pressure resistance, genomic stability, energy metabolism, and aging [4]. In mammals, seven sirtuin members have different enzymatic activities and functions, of which the least is known about SIRT4. SIRT4 is localized in the cytoplasm and has ADP-ribosyltransferase, aliphatic amidase and deacylase activities. Interestingly, the enzymatic activity and substrates of SIRT4 differ in different tissues and cells [5, 6]. Current studies have found that SIRT4 has separate oncogenic roles in different tumors [5, 7]. For example, SIRT4 promotes the progression of colorectal cancer [8]. In contrast, in hepatocellular carcinoma, overexpression of SIRT4 increased the survival of hepatocellular carcinoma cells under stressful conditions such as radiation [9]. Therefore, the role of SIRT4 in a specific type of tumor needs to be studied specifically. As a tumor with high malignancy and poor prognosis, there are no studies on the relationship between SIRT4 and BLCA.

In this study, we investigated the protein levels of SIRT4 in BLCA by immunohistochemical techniques on high-throughput tissue microarrays, analyzed the relationship between SIRT4 levels and clinicopathological parameters in BLCA patients and assessed the prognostic value. In addition, we selected the BLCA cell line T24 as a cellular model to analyze the effects of interference with and overexpression of SIRT4 on the proliferation, migration and invasion of BLCA cells in vitro. Moreover, we analyzed the effect of SIRT4 on the BLCA cell cycle and apoptosis. Mechanistically, we explored the relationship between SIRT4 and autophagy and its role in the inhibition of BLCA. Our results show that SIRT4 is an independent prognostic marker in BLCA and inhibits BLCA growth by suppressing autophagy.

## Results

### Low SIRT4 levels correlated with worse clinicopathological parameters and were an independent prognostic factor for BLCA

An increasing number of studies have shown that SIRT4 is involved in tumor development. However, the role of SIRT4 in bladder cancer has not been clarified. Additionally, in urological tumors, we recently reported that SIRT4 is critical for prostate cancer [10]. Therefore, we wanted to further explore the functional role of SIRT4 in bladder cancer.

**Fig. 1:**
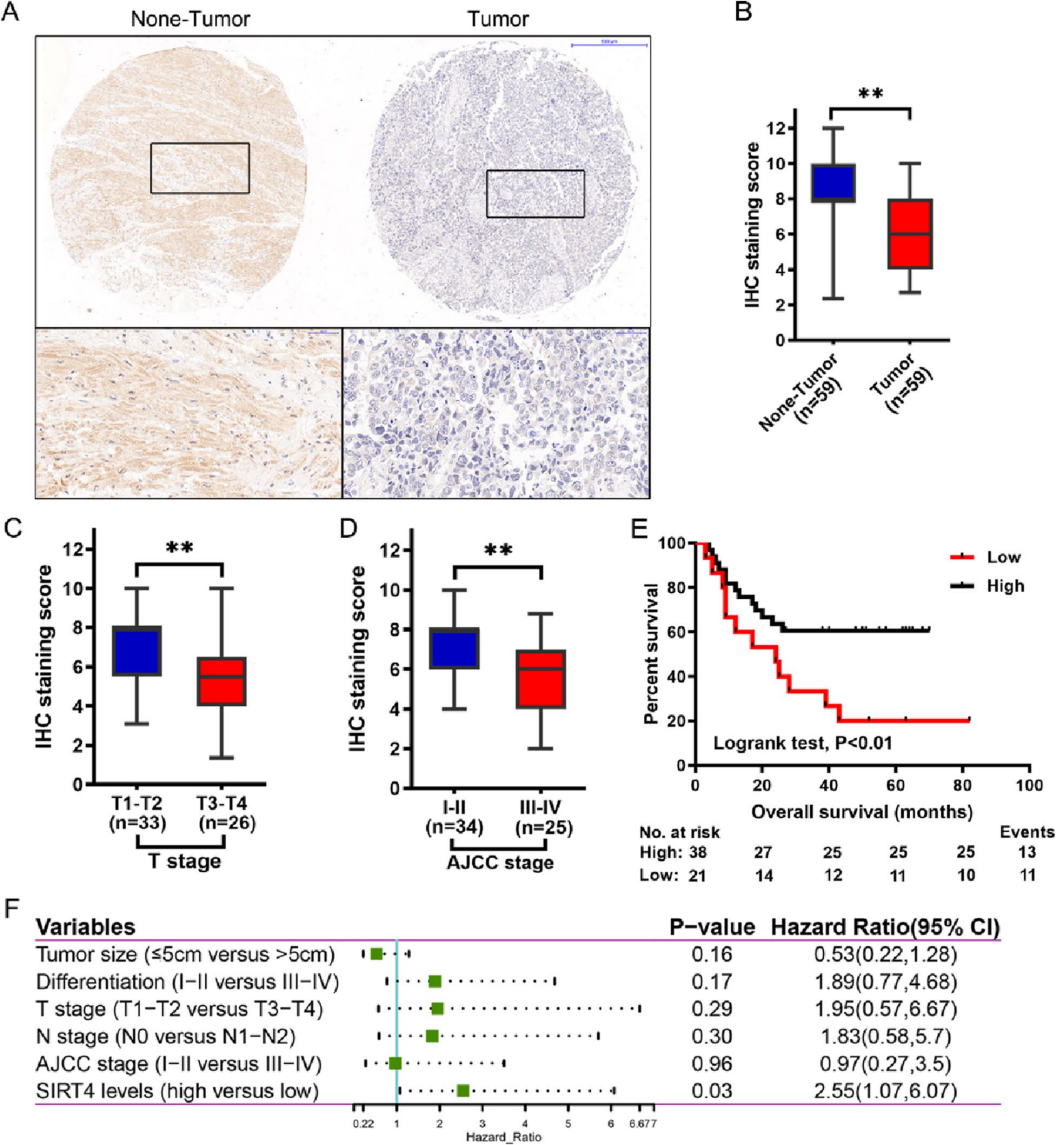
SIRT4 is downregulated in bladder cancer and is an independent prognostic factor for it. **A** SIRT4 is localized in the cytoplasm; SIRT4 staining is downregulated in tumor tissues compared to adjacent nontumorigenic bladder tissues. Paraneoplastic nontumor tissue is shown on the left, and tumor tissue is shown on the right. The lower images are magnified images in the box above. (Magnification: upper border 100× , lower border 400 ×). **B** Staining levels of SIRT4 in 56 BLCA tissues with 59 nontumor bladder tissues were analyzed by tissue microarray. SIRT4 protein levels were significantly reduced in tumor tissues compared with adjacent nontumorigenic bladder tissues (P < 0.01). Boxes represent interquartile range; whiskers represent 5th–95th percentile range; bars represent median. **C** The staining fraction of SIRT4 in different T-stages. It can be seen that the later the T-stage, the lower the staining score of SIRT4. **D** The staining fraction of SIRT4 in different AJCC stages. It can be seen that the later the AJCC stage, the lower the staining score of SIRT4. **E** Overall survival time of BLCA patients with low SIRT4 levels was significantly lower than that of patients with high SIRT4 levels by staining score analysis of immunohistochemistry (P < 0.01). **F** Cox multivariate analyses were performed on the overall survival time of BLCA patients. SIRT4 is the independent prognostic factor for BLCA patients

We first performed immunohistochemical staining of tissue microarrays containing 59 human BLCA tissues and 59 paraneoplastic nontumor bladder tissues, and we found that SIRT4 was mainly expressed in the cytoplasm (Fig. 1A) and that the staining level of SIRT4 was significantly lower in tumor tissues than in paraneoplastic nontumor bladder urothelial tissues (Fig. 1A, B). Next, we evaluated the relationship between SIRT4 levels and clinicopathological characteristics of patients with BLCA based on immunohistochemical staining results (Table 1). We found that low levels of SIRT4 staining were more likely to be seen in patients with larger tumor size, later T-stage (Fig. 1C), and later AJCC stage (Fig. 1D) (P values of 0.02, 0.02 and 0.02, respectively). We next further analyzed the factors influencing the prognosis of patients with BLCA. By log-rank test, we found that SIRT4 level, T stage and AJCC stage were factors affecting the overall survival time of BLCA patients (Table 2), and BLCA patients with low SIRT4 levels had worse overall survival time compared with those with high SIRT4 levels (P < 0.01) (Fig. 1E). To further corroborate whether SIRT4 is an independent prognostic factor in BLCA patients, we performed Cox regression analysis. We found that SIRT4 levels were independent prognostic factors for BLCA patients (Table 3) (Fig. 1F). Together, these results suggest that SIRT4 may be involved in the development of bladder cancer.

**Table 1:** Correlation between SIRT4 levels and clinicopathologic variables in bladder urothelial carcinoma

Clinicopathologic parameters	SIRT4 levels			*χ*^*2*^	*P* value^a^
	All cases	Low	High		
Age (years)				0.01	0.92
≤ 60	14	6	8		
> 60	45	20	25		
Gender				2.78	0.14
Male	50	20	30		
Female	9	1	8		
Tumor size (cm)				6.0	**0.02**
≤ 5	37	10	27		
> 5	22	13	9		
Differentiation				0.21	0.65
I-II	20	6	14		
III-IV	39	14	25		
Stage (T)				5.4	**0.02**
T1-T2	33	6	27		
T3-T4	26	12	14		
Stage (N)				0.01	0.92
N0	37	13	24		
N1-N2	22	8	14		
AJCC stage				5.5	**0.02**
I-II	34	10	24		
III-IV	25	15	10		

Bold values are statistically significant (*P* < 0.05)

^a^Chi-square test

**Table 2:** Univariate analysis of overall survival time in 59 patients with bladder urothelial carcinoma

Variable	All cases	Overall survival (months)		*χ*^*2*^	*P* value^a^
		Mean	Media		
Age (years)				0.12	0.73
≤ 60	14	36.7	23.0		
> 60	45	45.7	43.0		
Gender				0.01	0.98
Male	50	45.0	39.0		
Female	9	33.9	17.0		
Tumor size (cm)				0.47	0.50
≤ 5	37	37.4	25.0		
> 5	22	49.9	63.0		
Differentiation				2.80	0.09
I-II	20	48.9	40.0		
III-IV	39	38.6	24.0		
T stage				8.11	**0.01**
T1-T2	33	51.1	39.0		
T3-T4	26	31.5	17.0		
N stage				1.82	0.18
N0	37	47.2	43.0		
N1-N2	22	24.3	25.0		
AJCC stage				6.50	**0.01**
I-II	34	48.9	38.0		
III-IV	25	31.9	20.0		
SIRT4 levels				8.58	**0.01**
High	38	47.5	37.0		
Low	21	27.9	24.0		

Bold values are statistically significant (*P* < 0.05)

^a^log-rank test

**Table 3:** Cox multivariate analyses of prognostic factors for overall survival

Variables	HR	95% CI	*P* value^a^
Tumor size (cm) (≤ 5 versus > 5)	0.53	0.22–1.28	0.16
Differentiation (I-II versus III-IV)	1.89	0.77–4.68	0.17
T stage (T1-T2 versus T3-T4)	1.95	0.57–6.67	0.29
N stage (N0 versus N1-N2)	1.83	0.58–5.70	0.30
AJCC stage (I-II versus III-IV)	0.97	0.27–3.50	0.96
SIRT4 levels (high versus low)	2.551	1.07–6.07	**0.03**

Bold values are statistically significant (*P* < 0.05)

*HR* hazard ratio, *CI* confidence interval

^a^Forward:LR method

### SIRT4 inhibits BLCA cell growth, migration, and invasion

**Fig. 2:**
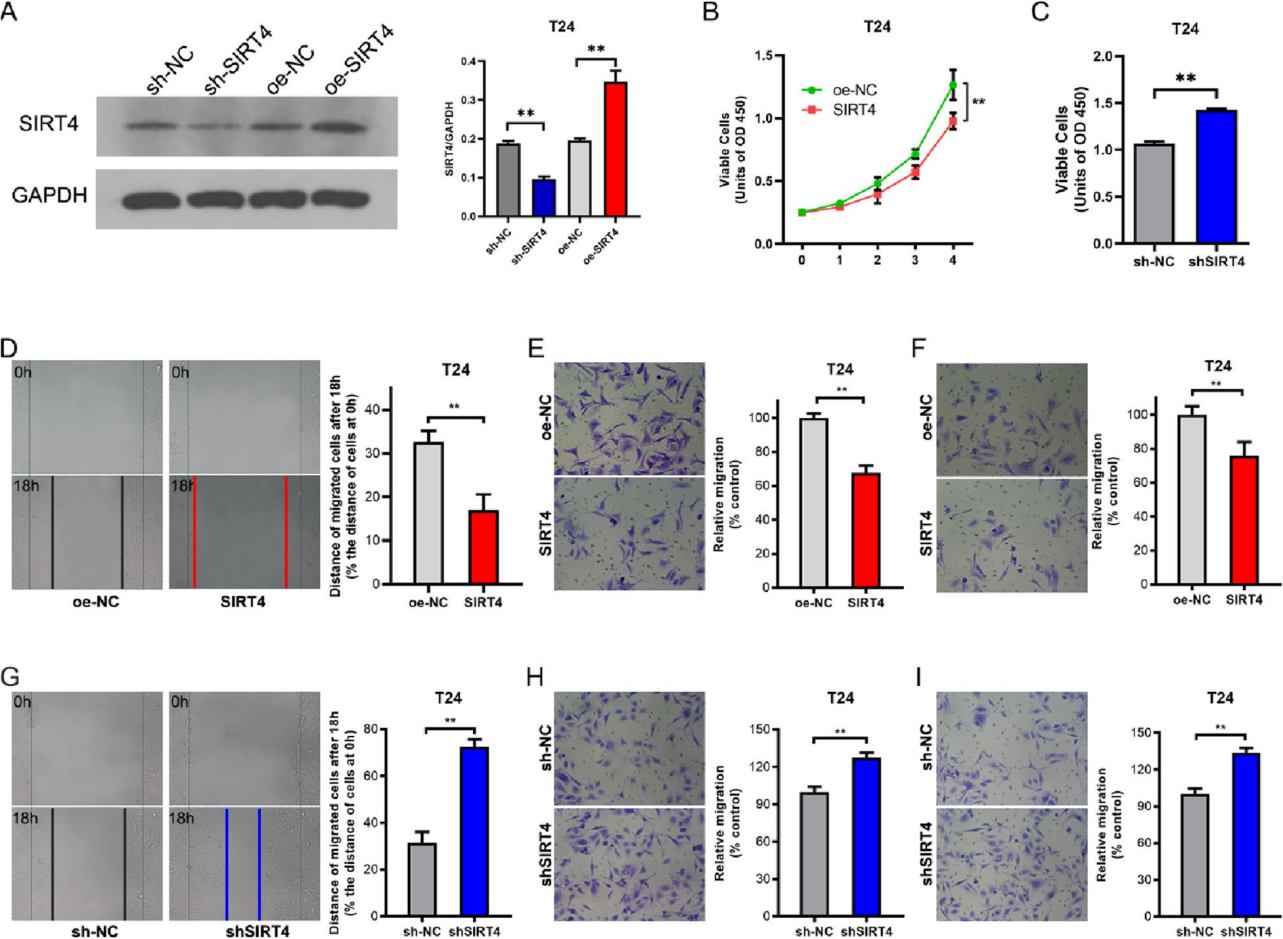
SIRT4 inhibits the proliferation, migration and invasion of BLCA cells in vitro. **A** T24 cells transfected with oeSIRT4 or shSIRT4, and the effect of overexpression or interferencing of SIRT4 was detected by Western blotting, with GAPDH as an internal control (Uncropped gel in supply Fig. 1). **B** and **C** Proliferation viability of T24 cells after overexpression (B) or interference (C) with SIRT4 was assayed with CCK-8 reagent. For figure C, cell proliferation viability was measured on the fifth day after seeding. **D** and **G** Effect of overexpression (D) or interfering (G) with SIRT4 on the scratch healing ability of T24 cells. Detection was performed after 24 h of serum-free culture after scratching. **E** and **H** Effect of overexpression (E) or interference (H) with SIRT4 on the migration ability of T24 cells. Counting was performed after taking random images of 5 areas in each well with a 400 × microscope. **F** and **I** Effect of overexpression (F) or interference (I) with SIRT4 on the invasive ability of T24 cells. Images of 5 areas were taken randomly in each well with a 400 × microscope after counting. *P < 0.05, **P < 0.01

We next evaluated the growth of BLCA in the presence of SIRT4 overexpression or interference (Fig. 2A). To our surprise, overexpression of SIRT4 significantly inhibited T24 BLCA cells, while interference with SIRT4 promoted growth (Fig. 2B, C). In addition, the wound healing rate of T24 cells was significantly decreased after SIRT4 overexpression (Fig. 2D), along with a significant decrease in cell invasion and migration ability (Fig. 2E, F). In contrast, however, interfering with SIRT4 significantly promoted the scratch healing ability (Fig. 2G), migration ability (Fig. 2H) and invasion ability (Fig. 2I) of T24 cells. Taken together, these data demonstrate that SIRT4 represses BLCA growth, migration and invasion.

### SIRT4 inhibits the cell cycle and induces apoptosis in BLCA

**Fig. 3:**
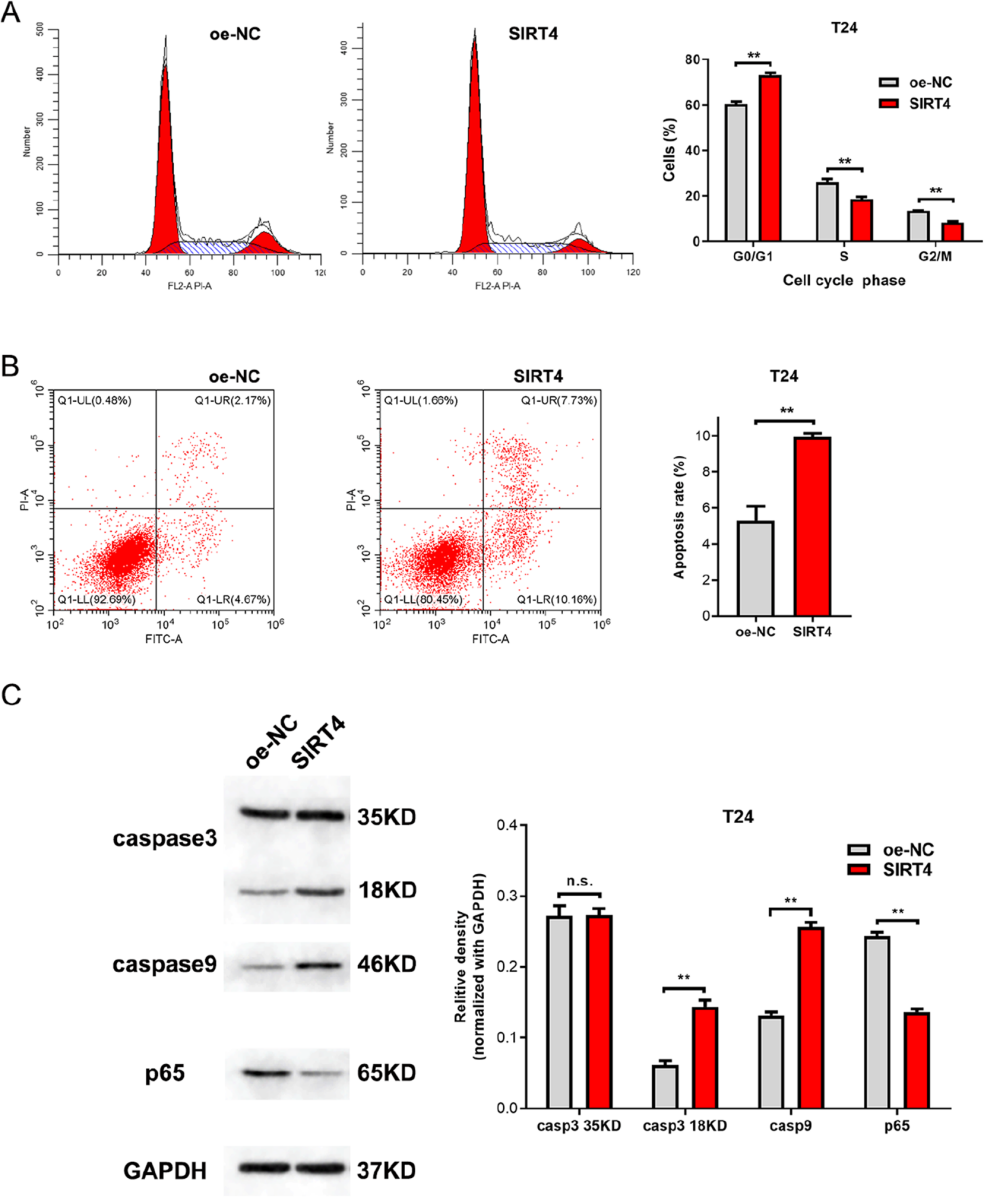
Overexpression with SIRT4 inhibit cell cycle and promotes apoptosis in T24 cells. **A** The effect of overexpression of SIRT4 on the cell cycle distribution of T24 cells was analyzed by PI Cell Cycle Assay Kit. Modfit (Verity Software House, Topsham, ME, USA). **B** Flow cytometry (C6; BD, Franklin Lakes, NJ, USA) to detect the effect of overexpression SIRT4 on apoptosis of T24 cells. APC and 7-AAD were used as staining agents. The statistical graph on the right calculates the percentage of stained by APC, i.e., early and mid-late apoptosis. **C** Effect of interference with SIRT4 on apoptosis-related proteins caspase 3, caspase 9 and p65 in T24 cells was detected by Western Blot. Band density was analyzed and quantified using ImageJ software (version 2.1.4.7; National Institutes of Health, Bethesda, MD, USA). *P < 0.05, **P < 0.01 (Uncropped gel in supply Fig. 2)

Our results suggest that SIRT4 may be an important gene in regulating BLCA growth. Next, to assess its role in BLCA survival, we examined the effects of SIRT4 on the cell cycle as well as apoptosis in T24 cells. We found that overexpression of SIRT4 significantly inhibited the cell cycle of T24 cells, with an increase in the proportion of G0-phase cells and a decrease in the proportion of S-phase and G2-phase cells (Fig. 3A). We next examined whether SIRT4 induced apoptosis in BLCA by Annex V and PI staining. As shown in Fig. 3B, the mean apoptotic rates of T24 cells before and after overexpression with SIRT4 were approximately 5 and 10%, respectively. In addition, we also investigated the changes in caspase-3, caspase-9 and p65 levels, which are important markers of apoptosis. The results showed that the levels of caspase-3 and caspase-9 in T24 cells were significantly increased after overexpression of SIRT4, while the levels of p65 were noticeably lower (Fig. 3C). These results show that SIRT4 inhibits the cell cycle of T24 cells and promotes apoptosis.

### SIRT4 inhibits the growth of BLCA by autophagy inhibition

**Fig. 4:**
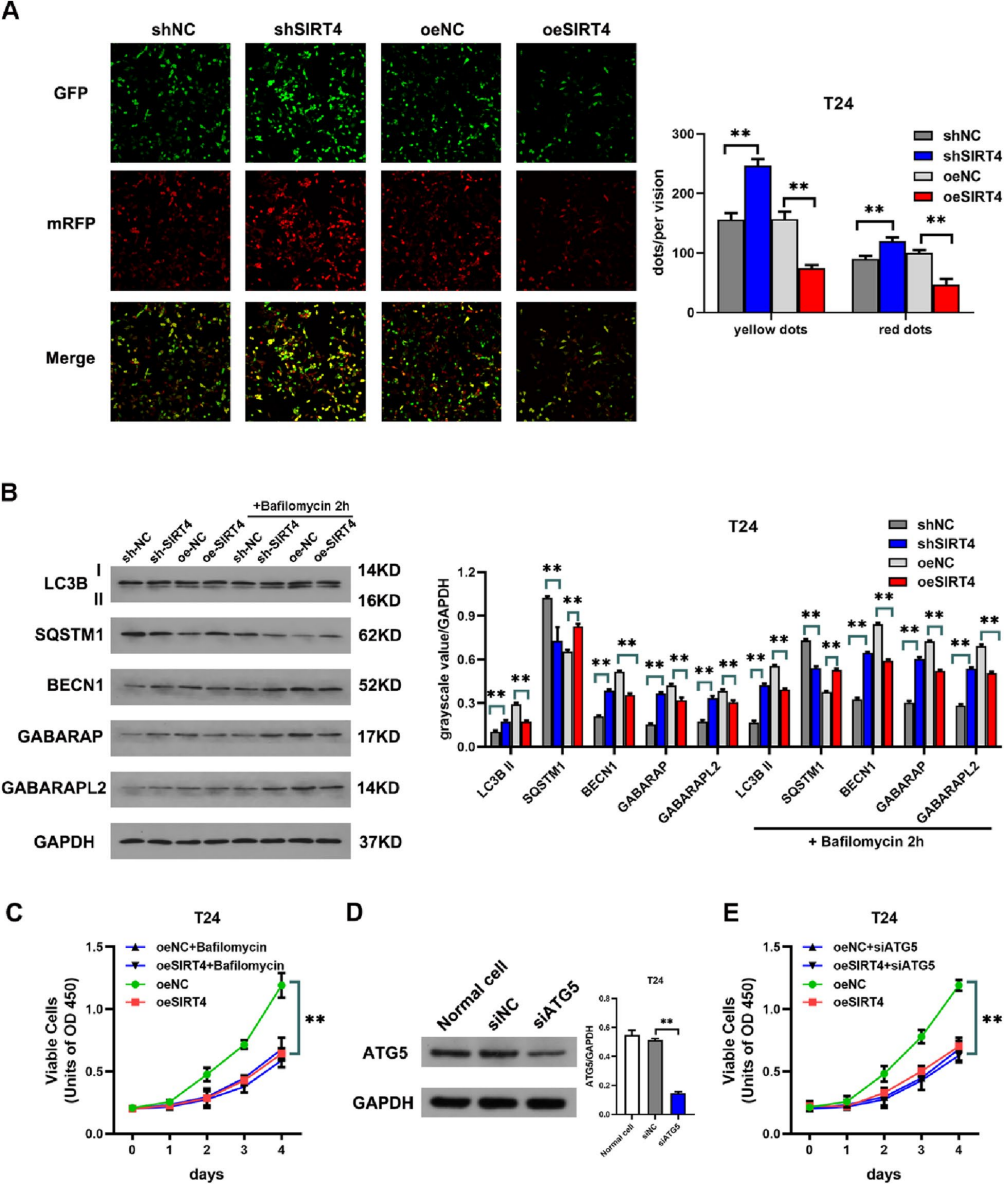
SIRT4 inhibits BLCA growth through autophagy inhibition. **A** RFP-GFP-tagged LC3 adenovirus was utilized to infect stably transfected T24 cell strains using SIRT4 overexpression and interference and negative control cells. Green dots indicate autophagosomes, yellow dots (marked in green and red) depict autophagic lysosomes without fusion with the lysosomes, and RFP signals without GFP relate to the fusion of autophagosomes with lysosomes. The yellow fluorescent particles within the cells were observed under the fluorescence microscope. **B** After 100 nmol/L bafilomycin treatment for 2 h, the changes in autophagy-related proteins LC3, SQSTM1, BECN1, GABARAP, and GABARAPL2 in T24 cells with SIRT4 overexpression and interference were detected (Uncropped gel in supply Fig. 3). **C** The inhibition effect on T24 cells with SIRT4 overexpression in the presence or absence of 100 nmol/L bafilomycins was detected using CCK-8. **D** Western blot analysis shows the interference effect of the shATG5 interference sequence on the ATG5 protein in T24 cells (Uncropped gel in supply Fig. 4). **E** In the presence or absence of shATG5, the inhibition effect on T24 cells with SIRT4 interference was detected by CCK-8

Autophagy is a phenomenon within which cell components are “cannibalized (self-eating)”; it degrades the contents of the package to achieve a dynamic process of cell homeostasis and organelle renewal [11]. It has been shown that autophagy is required for bladder cancer growth and survival [12]. Since recent evidence suggests that SIRT4 inhibits autophagy in colon cancer cells, we hypothesized that SIRT4 may regulate BLCA growth and survival by regulating autophagy. To test this idea, we assessed whether SIRT4 could inhibit the level of autophagy in BLCA cells. We infected T24 cells with RFP-GFP-tagged LC3 adenovirus and then observed the fluorescence signal by fluorescence microscopy. The green fluorescence was burst by acid, while the red fluorescence was stable to acid. Therefore, green fluorescence indicates autophagosomes, yellow dot fluorescence (overlapping green and red fluorescence) indicates autophagosomes that are not degraded by lysosomes, and the RFP signal without GFP indicates autophagic lysosomes. Thus, the red fluorescence intensity represents the level of autophagic flow. We found that red fluorescence and yellow fluorescence were significantly reduced in T24 cells after overexpression of SIRT4, while interference with SIRT4 enhanced the intensity and amount of red fluorescence and yellow fluorescence (Fig. 4A). This suggests that SIRT4 inhibits autophagic flow in BLCA cells. To further confirm the inhibitory effect of SIRT4 on BLCA autophagy, we evaluated the effect of SIRT4 on the levels of several classical autophagy markers. Overexpression of SIRT4 significantly decreased the levels of LC3B II, as well as BECN1, GABARAP, and GABARAP L2, while significantly increasing the levels of P62, while interference with SIRT4 obtained the opposite result (Fig. 4B). In the presence of the lysosomal inhibitor bafilomycin A1 (BFA), we still observed the same results. Taken together, these results suggest that SIRT4 inhibits BLCA autophagy.

It has been shown that inhibition of autophagy attenuates the tumor proliferation of BLCA cells. To investigate the role of autophagy inhibition in the inhibition of BLCA growth by SIRT4, we added the autophagy inhibitor bafilomycin A1 to the culture medium and then assayed the proliferation ability. We found that overexpression of SIRT4 in the presence of bafilomycin A1 no longer had a further inhibitory effect on BLCA cells (Fig. 4C). To further investigate whether SIRT4 inhibits BLCA proliferation by suppressing autophagy, we interfered with ATG5, a key gene for autophagy (Fig. 4D), with adenovirus and found that SIRT4 also no longer had further inhibitory effects on BLCA after interfering with ATG5 (Fig. 4E). Taken together, these findings suggest that inhibition of autophagy contributes to the effect on SIRT4 in BLCA.

## Discussion

This study, for the first time, analyzed the protein level of SIRT4 in BLCA and its relationship with clinicopathological parameters and survival time in BLCA patients. We found that SIRT4 was reduced in BLCA and associated with worse AJCC stage and was an independent prognostic factor in BLCA patients. Furthermore, we further found that overexpression of SIRT4 at the cellular level inhibited the proliferation and migratory capacity of BLCA cells, while interference with SIRT4 had the opposite effect. Moreover, we found that SIRT4 inhibited the cell cycle and increased apoptosis in BLCA cells. Mechanistically, we found that SIRT4 inhibits the growth of BLCA by suppressing autophagy. In conclusion, our study shows that SIRT4 is involved in the development of BLCA and that SIRT4 acts as a tumor suppressor in BLCA and is a potential prognostic marker and therapeutic target.

Interestingly, SIRT4 has previously been reported to function as both a tumor oncogene and a tumor suppressor gene. SIRT4 expression is downregulated in most tumors, including gastric cancer [13], colorectal cancer [8, 14], hepatocellular cancer [15] and thyroid cancers [16]. For example, Huang et al. found that SIRT4 was downregulated and correlated with worth pathological differentiation in gastric cancer [13]. In colorectal cancer, Huang et al. [8] and Miyo et al. [14] found that SIRT4 levels were reduced in patients with colorectal cancer and were associated with poor prognosis; moreover, SIRT4 inhibited the proliferation of colon cancer cells. However, some literature has also found that SIRT4 is upregulated in some tumors and that SIRT4 was found to have oncogenic effects. For example, SIRT4 was found to be upregulated in esophageal cancer [17], and overexpression of SIRT4 increased the clonogenic ability of hepatocellular carcinoma HepG2 cells in response to radiation [9]. In this study, SIRT4 levels were found to be reduced in BLCA tissues by tissue microarray and correlated with some pathological parameters of the malignant phenotype and were an independent prognostic factor in BLCA patients. At the same time, we selected the BLCA cell line T24 as experimental subjects and found that overexpression of SIRT4 significantly inhibited, while interference with SIRT4 induced, their proliferation as well as their migration ability in vitro. Furthermore, we found that overexpression of SIRT4 resulted in inhibition of the cell cycle and increased apoptosis in T24 cells. Our study reveals that SIRT4 plays a tumor suppressor role in BLCA.

Apoptosis deficiency can affect tumorigenesis, progression and metastasis [18]. The caspase family plays a crucial role in the death of apoptotic cells. Caspase-9 participates in the apoptosis process as an initiator caspase [19], while caspase-3 is a downstream protease of caspase-9 in the apoptotic pathway. In our experiments, we explored the relationship between SIRT4 and apoptosis. We found that the apoptosis rate of T24 cells increased after overexpression of SIRT4 by flow cytometry and found that overexpression of SIRT4 promoted elevated cleaved caspase-3 and caspase-9 and inhibited p65 levels by Western blot assay. Our results suggest that overexpression of SIRT4 may affect proliferation by promoting apoptosis of BLCA cells.

It is particularly important to note here that SIRT4 level, T stage and AJCC stage were all prognostic factors for bladder cancer in our univariate survival analysis, whereas the multifactorial analysis showed that SIRT4 was an independent prognostic factor. This result does not mean that T stage and AJCC stage are not important for the prognosis of bladder cancer, and there may be two reasons here. The first possibility is that because SIRT4 levels were correlated with T stage and AJCC stage, when a multifactorial prognosis was performed, T stage and AJCC stage may have been weakened in importance because they were both associated with SIRT4. The second possibility is that because the sample we used was not very large, there was some selection bias that caused T stage and AJCC stage to be less significant in the multifactorial prognostic analysis. However, the univariate survival analysis of T stage and AJCC stage still showed a significant correlation with prognosis, which is in line with our general perception. We await further expansion of the sample to validate this result in the future.

Autophagy is considered to be a self-protective mechanism during tumor growth because autophagy engulfs cellular debris by waste utilization to generate energy to meet the exuberant energy demands of tumor cells [20]. Similar to many autophagy-related genes, the expression and function of the SIRT family are affected by intracellular stress and nutritional conditions [21]. For other members of the SIRT family, knockdown of the SIRT1 gene was found to disrupt or eliminate autophagy in mammalian cells [22]. SIRT1 regulates autophagy by deacetylating autophagy-associated proteins or regulating the binding sites of transcription factors to the promoter regions of autophagy-associated genes [23]. Both SIRT2 and SIRT5 have also been reported to be associated with autophagy [24, 25]. Our recent study showed that SIRT4 enhances the sensitivity of colon cancer cells to 5-FU by inhibiting autophagy (not published). In addition, others found that SIRT4 can inhibit autophagy through the Akt/mTOR pathway [26], and SIRT4 depletion can activate the autophagic process in MGCs by regulating the AMPK-mTOR signaling pathway [27]. However, in pancreatic ductal adenocarcinoma (PDAC), it has been reported that SIRT4 inhibits tumor growth and promotes autophagy [28]. Until recently, the relationship between SIRT4 and autophagy has not been investigated in bladder cancer. In the present study, we found that SIRT4 inhibited autophagic flow in T24 cells by RFP-GFP-tagged LC3 adenovirus, a result that was further validated in several autophagy-related markers. The growth inhibition of T24 cells caused by overexpression of SIRT4 was counteracted when autophagy was inhibited by drugs or by interfering with autophagy-related genes. Our results suggest that SIRT4 inhibits the growth of bladder cancer cells by suppressing autophagy. Because the role of autophagy in cancer is context dependent, as a tumor suppressor gene, autophagy may function in some tumors through inhibition of autophagy and in others through activation of autophagy. Mechanistically, it was found that in PDAC, SIRT4 promotes autophagy by inhibiting glutamine metabolism and further activating the phosphorylation of p53 [28]. However, glutamine metabolism produces ammonia, which induces autophagy, so it is also possible that SIRT4 inhibits ammonia production by suppressing glutamine metabolism, thereby inhibiting ammonia-induced autophagy. This is a very interesting topic to further study.

In summary, the present study suggests that SIRT4 immunohistochemistry may be a reliable prognostic biomarker in BLCA and that SIRT4 plays the role of tumor suppressor in BLCA. Thus, SIRT4 may represent a promising target for the diagnosis and treatment of BLCA.

## Methods

Given the retrospective nature of the study, ethical approval was granted by the local ethics committee of the Shanghai XinChao Biotechnology Co (Shanghai, China). The study was conducted in accordance with the principles of the Declaration of Helsinki.

### Patient and tissue samples

For this study, 59 cases of individual patient samples were included. The age range of the patients was 44 to 85 years of age with a mean age of 68 years. Patients did not receive preoperative chemotherapy or radiotherapy before surgery. The clinicopathological parameters included the patient’s age, sex, tumor size, pathological grade, depth of tumor invasion (T), lymph node status (N), and clinical staging (the eighth edition of the American Cancer Federation staging system was used for TNM staging). Clinicopathological parameters are shown in Table 1.

Tissue gene array chips were obtained commercially (Superchip Inc., Shanghai, China) and included 59 BLAC samples and corresponding adjacent nonneoplastic tissue specimens. Thus, one tissue microarray included 118 points. The diameter of the tissue on the tissue microarray was 1.5 mm, and all samples were coated with paraffin wax.

### Immunohistochemistry

A tissue microarray was prepared by baking the array in a hot incubator for 2 h, after which it was incubated twice in xylene for 5 min at 37 °C to deparaffinize the specimen. To rehydrate the specimen, the tissue microarray was transferred every 5 min to sequentially graded ethanol concentrations at 100%, 100%, 95%, 85%, and 70%. Antigen retrieval was performed in a pressure cooker in citrate buffer (10 mM citrate and 0.05% Tween 20, pH 6.0). To suppress endogenous peroxidase activity, the tissue microarray was incubated in 0.3% H_2_O_2_ in Tris–HCl buffer for 15 min at 37 °C. Subsequently, the tissue microarray was incubated overnight with a polyclonal rabbit anti-SIRT4 antibody (HPA029692, 1:400, Sigma, USA) at 4 °C. A secondary antibody was applied using the GTVision Kit (Gene Tech Inc., Shanghai, China). Next, the microarray chip section was stained with a diaminobenzidine (DAB) solution and counterstained with hematoxylin. The chip was then dehydrated and sealed with a coverslip. As a negative control, tissue was treated with antibody dilution solution alone.

After the tissue microarray staining was completed, the chip was imaged by scanning through a scanner (3D HISTECH, Budapest, Hungary), and a file was created that contained all the tissue information on that tissue section. The file can be magnified 1–400 times in the Pannoramic software (3D HISTECH, Budapest, Hungary), and two pathologists who were unaware of the patient's information used the software on their computers to score the degree of staining. Each tissue point was assigned a score that was based on the staining intensity multiplied by the area of the staining [8]. The staining intensity was divided into four categories and included the following criteria: 0 = no staining; 1 = weak staining; 2 = moderate staining; and 3 = strong staining. The staining area assessment was as follows: 0 = 5% or none of the cells were stained; 1 = 5–25% of the cells were positively stained; 2 = 26–50% of the cells were positively stained; 3 = 51–75% of the cells were positively stained; and 4 = more than 75% of the cells showed positive staining. The final degree of staining was divided into two categories: 0–6 = low expression and 7–12 = high expression. When the evaluation of the staining patterns did not match, a consensus opinion was performed by both pathologists.

### Cell lines and culture conditions

The human BLCA cell line T24 was purchased from Shanghai Institute of Cell Biology, Chinese Academy of Sciences, in October 2021. The cell bank used four primer pairs, DXS52, MD17S5, Apo-B and D2S44, to monitor changes in the cell lines during passage. The cells were last tested in October 2020, and the experiment ended 5 months after the cells were purchased. The cells were maintained in Dulbecco’s modified Eagle’s medium (DMEM; Gibco, USA) supplemented with 10% fetal bovine serum (Gibco, USA) and penicillin/streptomycin (Gibco, USA). The conditions for the CO2 incubator were 37 °C and 5% CO2.

### Vector and virus production

We designed three siRNA sequences against SIRT4, namely, 5′-GCGTGTCTGAAACTGAATTCT-3′, 5′-GCTCCTGATGGTGACGTCTTTCTCT-3′ and 5′-GCGTTCAATGTGGAGGCCATCTGAA -3′. The negative control sequence was 5′-TTCTCCGAACGTGTCACGTAA-3′. The following are the ATG5 interference sequences: ATG5-HOMO-853: 5′-CCAUCAAUCGGAAACUCAUTT-3′, ATG5-HOMO-393: 5′-GACGAAUUCCAACUUGUUTT-3′, and ATG5-HOMO-940: 5′- GACCUUUCAUUCAGAAGCUTT-3′, and the negative control sequence is 5′-UUCUCCGAACGUGUCACGUTT-3′. Lentiviruses that overexpressed and silenced SIRT4 were purchased from Hanbio (Shanghai, China), and the lentiviral vector was pHBLV-U6-Puro. The final titer of lentivirus and negative control virus was 2 × 10^8^ PFU/ml. We screened the siRNA sequences with the highest interference efficiency by PCR experiments (data not shown). Cells were then transfected with the overexpressing or interfering lentivirus with the highest interference efficiency for 72 h and screened with puromycin to achieve stable transfection of the overexpressing or interfering SIRT4 lentivirus in T24 cells.

### Reverse transcription RT‒PCR

Total RNA from the tissue was purified using the TRIzol kit (Invitrogen, USA) following the manufacturer’s protocol. Total cDNA (500 ng) was synthesized using a reverse transcription kit (PrimeScriptTM RT Master Mix, TaKaRa, Japan). The cDNA was diluted 3 times using the RT‒PCR Kit (SYBR^®^ Premix Ex Taq^™^ II, TaKaRa, Japan) in the RT‒PCR apparatus (DNA engine option 2, Bio-Rad). GAPDH was selected as the reference gene. Primers for each gene were as follows: SIRT4 forward primer 5′-GCGAGAAACTTCGTAGGCTG-3′, reverse primer 5′-TCAGGACTTGGAAACGCTCT-3′; GAPDH forward primer 5′- TCAAGAAGGTGGTGAAGCAGG-3′, reverse primer 5′-TCAAAGGTGGAGGAGTGGGT-3′. The PCR conditions were as follows: 2 min at 94 °C, 30 s at 94 °C, 30 s at 57 °C, 1 min at 72 °C for 40 cycles, 5 min at 72 °C and cooling at 4 °C. After the loop, the melting curve was analyzed to ensure uniformity of the PCR product. Gene expression was calculated by the 2^−∆∆^Ct method.

### Western blot

Cells were lysed with RIPA lysis buffer (Beyotime, China) supplemented with a protease inhibitor cocktail (Beyotime, China). Protein concentrations were determined by a BCA protein concentration reagent kit (Beyotime, China). Cell lysates were separated by SDA-PAGE and transferred to PVDF membranes. The reference antibodies for this assay were rabbit anti-human SIRT4 (36 kDa) polyclonal antibody (HPA029692, Sigma, USA), rabbit anti-human caspase3 (35/18 kDa) polyclonal antibody (9662, CST, USA), rabbit anti-human caspase9 (46/35 kDa) polyclonal antibody (10380-1-AP, Proteintech, China), rabbit anti-human p65 (65 kDa) polyclonal antibody (10745-1-AP, Proteintech, China), abbit anti-human LC3B monoclonal antibody (ab192890, Abcam, England), rabbit anti-human p62 monoclonal antibody (ab109012, Abcam, England), rabbit anti-human beclin1 monoclonal antibody (ab207612, Abcam, England), rabbit anti-human GABARAP monoclonal antibody (ab109299, Abcam, England), rabbit anti-human GABARAPL2 monoclonal antibody (ab126607, Abcam, England), rabbit anti-human ATG5 monoclonal antibody (ab108327, Abcam, England), goat anti-rabbit detection antibody (ab97200, Abcam, England), and rabbit anti-human GAPDH (37 kDa) polyclonal antibody (AB-P-R 001, Goodhere, China).

### Cell proliferation activity

Cells were seeded at 1000/well for cell proliferation activity in a 96-well plate. The medium was then changed every other day and incubated until the day when the assay was performed. For the assay, 10 µl of CCK-8 (Dojindo, Japan) solution was added to each well, and then the absorbance was measured at 450 nm after incubation in a CO2 incubator for 2 h.

### Wound healing assay

The experimental group and negative control group cells of T24 cells were seeded in 5*10^5^ per well in 6-well plates and cultured overnight. When the cell density reached approximately 90%, a 200 µl tip head was placed perpendicular to the bottom of the 6-well plate. The cells were washed 3 times with PBS and then incubated in serum-free medium for 24 h. Images of cells after scraping and at the end of culture were obtained at 3 locations using a light microscope, which was used to calculate the distance of cell migration.

### Cell migration and invasion assays

For the migration assay, the transfected cells (6 × 10^4^ cells) were suspended in 0.2 ml of serum-free medium and seeded in the upper chambers of 24-well Transwell plates (Corning Inc., Corning, NY, USA). The lower chambers were filled with 0.6 ml of growth medium containing 10% FBS. Cells were cultured at 37 °C and allowed to migrate for 18 h. After migration, cells in the top chambers were removed with a cotton swab, and the cells in the bottom chambers were fixed in 4% paraformaldehyde and stained with 0.1% crystal violet (Sigma, USA). Stained cells were counted under the microscope in five random fields of view of each well (Olympus, Japan). For the invasion assay, a similar protocol was followed except that the top chamber of the transwell plate was precoated with Matrigel (BD Biosciences).

### Flow cytometric analysis of the apoptosis rate and cell cycle

Cells were harvested by trypsinization, pelleted by centrifugation, and resuspended in PBS containing 3% fetal bovine serum. The measurement of cell apoptosis was performed by flow cytometry (C6, BD, USA) using annexin V-APC and 7-AAD (BD, USA) staining. The apoptosis rate was calculated for early apoptosis, which were the cells stained by APC. Flow cytometry analysis software was Accuri C6 software (BD, USA). The cell cycle was determined using a propidium iodide (PI)/RNase kit (BD, USA), and the results were examined with the ModFit analysis software program (Verity Software House, Topsham, ME, USA).

### Confocal fluorescence microscopy

Cells were cultured on glass slides, transiently transfected with the GFP-RFP-LC3 adenovirus, and processed accordingly. After being washed with PBS buffer, the cells were fixed with 4% paraformaldehyde. The slides were then sealed with glycerol, and the location of the LC3 spot was observed using a confocal fluorescence microscope.

### Statistical analysis

Statistical analysis was performed using the SPSS software package version 20.0 (SPSS, Inc., IBM, USA). Data from three or more independent experiments are expressed as the mean ± standard deviation. A paired Student’s t test was used to analyze the final score of the tumor and nontumor tissues. Chi-squared analysis was used to analyze the relationship between SIRT4 horizontal grouping and clinicopathological parameters. The Kaplan‒Meier method (the log-rank test) was used for single-factor analysis, and the Cox proportional hazards regression model was used to identify independent prognostic factors. *P* < 0.05 (two-tailed) was considered statistically significant.

## Data Availability

The datasets used and/or analyzed during the current study are available from the corresponding author on reasonable request.
